# Pupillometry for pain assessment in noncommunicating children in the pediatric intensive care unit: a prospective accuracy study

**DOI:** 10.1007/s00431-026-07004-3

**Published:** 2026-05-02

**Authors:** A Portefaix, M Rabilloud, F Baudin, E Dantony, T Ginhoux, B Kassaï-Koupaï, E Javouhey, F Bordet

**Affiliations:** 1 Clinical Investigation Center INSERM P-1407, Hospices Civils de Lyon, Lyon, France; 2 RESHAPE U1290 Unit, Université Claude Bernard Lyon 1, Lyon, France; 3 Laboratoire de Biométrie et Biologie Evolutive UMR 5558, Université Lyon 1, CNRS, 69100 Villeurbanne, France; 4 Service de Biostatistique Et Bioinformatique, Hospices Civils de Lyon, Pôle Santé Publique, 69003 Lyon, France; 5 Service de Reanimation Pédiatrique, Hospices Civils de Lyon, Hopital Femme Mère Enfant, 69500 Bron, France; 6 APCSE (Agressions Pulmonaires et Circulatoires dans le Sepsis), VetagroSup, Université de Lyon, Marcy-L’Étoile, 69280 France; 7 EA 7426 Joint Research Unit HCL-bioMérieux, Université Claude Bernard Lyon 1, Villeurbanne, Auvergne-Rhône-Alpes France; 8 Laboratoire Interdisciplinaire d’étude du Politique–Hannah Arendt, LIPHA (EA7373), Université Gustave Eiffel, Champs-Sur-Marne, France

**Keywords:** Pediatric intensive care unit, Analgesia assessment, Pupillometer, COMFORT-B

## Abstract

Pain assessment in noncommunicating children in pediatric intensive care unit (PICU) remains a significant clinical challenge, particularly in deeply sedated patients. Pupillometry, a noninvasive objective technique, showed promise in anesthesia settings, but its relevance in PICU is unclear. We assessed feasibility and diagnostic performance of video pupillometry for pain assessment in noncommunicating intubated children in PICU. We conducted a prospective, single-center diagnostic accuracy study in the French PICU of Lyon (France). Children aged 0–18 years under mechanical ventilation and sedation were eligible. Pupillary diameter variation was measured using the AlgiScan® video pupillometer. Pain was defined as a COMFORT-B score > 17, assessed by nurses blinded to pupillometry results. Diagnostic performance was evaluated using the area under the ROC curve (AUC). Specificity was estimated at a sensitivity threshold of 90%. Sixty-six patients were included and 231 individual measurements analyzed; 52 patients (85%) had at least one complete series. The AUC was 73.6% [95% CI 61.1–82.6]. A weak correlation between COMFORT-B scores and pupillary diameter variation (Spearman’s *ρ* = 0.4, *p* < 0.05) was found. At 90% sensitivity, the threshold was 0.145, with a specificity of 52.2%. Feasibility was limited in neonates under 5 kg and agitated children.

*Conclusion*: To ensure a sensitivity at 90%, the corresponding specificity was quite low and correlation with COMFORT-B was also weak. Feasibility in young infants remains limited due to technical constraints. Further multicenter studies are warranted to clarify its role in analgesia assessment in PICU, as an adjunct to other techniques.

*Trial registration*: ClinicalTrials.gov identifier: NCT02847195.

**Table:** 

**What is Known:** • *Pain assessment in noncommunicating children in the pediatric intensive care unit (PICU) remains a major clinical challenge, particularly in sedated or critically ill patients. Additional tools are needed to better assess pain in this population.*	
**What is New:** • *A weak correlation was found between pupil variation measured by video pupillometry and the COMFORT-B score in PICU patients. Pupillometry may complement existing tools as part of a multimodal approach to pain assessment in this population.*

**Supplementary Information:**

The online version contains supplementary material available at 10.1007/s00431-026-07004-3.

## Introduction

Pediatric intensive care interventions are frequently painful requiring sedation and analgesia. Accurate pain assessment is particularly challenging in noncommunicating children (intubated or deeply sedated notably). A systematic review revealed that only 58% of sedated children in pediatric intensive care units (PICUs) received optimal sedation, 10% were undersedated and 32% oversedated [1].

Several tools are available for the assessment of analgesia: observational scales such as the COMFORT-B score, physiological parameters like heart rate and blood pressure, and neurophysiological monitoring tools such as the Bispectral Index (BIS). Among these, the COMFORT-B scale is the most commonly used and recognized as the gold standard in PICUs [2–4]. However, it presents limitations, particularly in its ability to discriminate between the effects of sedation and those of analgesia.

Pupil diameter measurement has been used in anesthesia to evaluate analgesic depth. This can be assessed visually or more precisely through an automated pupillometer, a noninvasive medical device that offers accurate and reproducible measurements [5, 6].

In adult surgical patients, studies have shown a correlation between increased analgesia depth and reduced pupil diameter [5]. In children sedated with sevoflurane in the operating room, pupil dilation is an earlier indicator of pain than physiological parameters [7–9]. Furthermore, in postoperative pediatric patients undergoing pectus excavatum surgery, a correlation has been established between Visual Analog Scale (VAS) pain scores and pupil dilation: Each one-point increase in VAS corresponds to a 0.39% increase in pupil diameter [10]. Moreover, opioids reduce pupil dilation in response to pain in proportion to their blood concentration [11]. In adult intensive care unit, pupillometry has been evaluated, with inconsistent results [12–14]. The use of various protocols on the video pupillometer, such as the Pupillary Pain Index (PPI) or tetanic stimulation, has discordant results regarding the correlation between pupillary diameter variation and pain perception [15, 16]. Reported diagnostic performance varies widely, with specificity reaching up to 100% and sensitivity up to 71% [14].

However, in PICU, sedation and analgesia practices are less standardized than in adult intensive care unit, with variability in sedation depth as well as in pharmacological strategies. In contrast to the operating room setting, withdrawal syndrome and agitation are frequent in PICU. All these factors contribute to making pain assessment particularly complex in this context [17, 18].

To date, pupillometry has not been studied in PICU. This prospective study aims to evaluate its performance in this context and to investigate the correlation between pupil diameter and COMFORT-B score [2, 3]. Our secondary objectives were to assess the correlation between pupillary diameter variation and the COMFORT-B score in sedated children and to describe the feasibility of video pupillometry.

## Methods

We performed a prospective monocentric study in the pediatric intensive care unit of Lyon Children-Mother Hospital (France). This trial, registered in https://www.clinicaltrials.gov (NCT02847195), was sponsored by the Hospices Civils of Lyon (HCL). The Clinical Investigation Center (INSERM P-1407), part of the HCL, coordinated the trial and collected all data. The trial was approved by the French Southeast IV Ethics Committee (2014–026-2) in November 2014, registered by the French National Agency for Medicines and Health Products Safety (ANSM ID RCB: 2013-A01600-45) in November 2013, and declared to French data protection authority (CNIL declaration #1,814,110) in December 2014.

A written informed consent from both parents/legal representatives was required before any procedure of the study. This manuscript adheres to the applicable STARD guidelines.

### Patients

Inclusion criteria were children aged 0 to 18 years, hospitalized in the PICU with mechanical ventilation and sedation, and with parental consent.

Noninclusion criteria were administration of neuromuscular blocking agents (which prevent COMFORT-B assessment), acquired or congenital neurological disorders, ophthalmologic conditions, and lack of health insurance coverage.

Patients were included during daytime hours (8:00 a.m. to 8:00 p.m.), according to physician availability.

### Measures

Pupillary diameter variations were measured using the AlgiScan® pupillometer (ID-Med, Marseille, France, instructions for use in Annex 1), in pupillary reflex dilation (PRD) mode. Pupillary variation was recorded over a 10-s period. Measurements were performed at three time points for each patient: before, during, and after a standardized painful procedure (tracheal suctioning), and for each of the two eyes, leading to a maximum of six recordings per patient for each measurement time. Each patient could undergo up to three measurement series during hospitalization (i.e., up to 18 recordings per patient in total).

Details of the measurement series are presented in the Supplementary Material (Figure S1).

Tracheal suctioning was selected as it is a routine component of patient care in the PICU, enabling the assessment of nociceptive responses without introducing an additional painful stimulus. In our unit, nurses follow a standardized protocol for tracheal suctioning. Preoxygenation is administered for 3 min prior to, and for 2 min following, the procedure. The first suctioning is performed without instillation of saline; subsequent suctioning is performed with saline. The maximum insertion depth of the suction catheter into the endotracheal tube is determined according to standard reference charts available in each patient room. To reduce evaluation bias, the COMFORT-B score was recorded concurrently by the patient’s nurse, blind to the pupillometry results, which were performed by a physician.

We chose to evaluate pain and sedation using the COMFORT-B score (Annex 2 in Supplementary), as it is validated for all pediatric age groups and is recommended by the European Society of Paediatric and Neonatal Intensive Care [2, 19].

Sedation levels were defined as follows: deep sedation for a COMFORT-B score between 6 and 10, adequate (or standard) sedation between 11 and 17, and a score above 17 indicating either awakening, discomfort, or pain. The sedation objective in PICU is most often to maintain a COMFORT-B score below 17.

Failures were defined as follows:

- Complete failure: inability to obtain any of the three scheduled measurements.
- Partial failure: inability to obtain one or two of the three scheduled measurements.

### Statistical analysis

The absolute variation in pupillary diameter between the pain period and the nonstimulated period was defined as the difference between the maximal and minimal pupillary diameters.

The sample size was determined for an area under the ROC curve (AUC) of 0.7 which was the minimum expected value and using the method described by Zhou et al. [20]. For a mean number of measurements per patient of six, an intraclass correlation (ICC) of 0.17 and a ratio of the number of measurements without pain on the number of measurements with pain equal to 8, the inclusion of 91 patients allowed to obtain a precision of the AUC estimate of 0.1 for a 95% confidence interval (CI) [21]. The ICC and the ratio were calculated using previous data corresponding to 204 measurements on 34 patients.

For a given patient and a given measurement time, if diameter variation (difference between the maximum and the minimum of the pupil diameter) was available for both eyes, the maximum of this variation was retained. To estimate the global diagnostic performance of the diameter variation, a nonparametric ROC curve was built considering COMFORT-B as the gold standard (pain being defined as a COMFORT-B score strictly superior to 17). The AUC was estimated using the De Long method [22]. In order to take account of the correlation between the measurement of a given patient, the 95% CI of the AUC estimate was calculated using a clustered bootstrap method with resampling of patients. To ensure a stable number of events (pain) in each bootstrapped sample close to the observed number, the sampling was stratified on the existence or not of at least one measurement with pain. Limits of the 95% CI were defined using the BCa (adjusted bootstrap percentile) method. Specificity was estimated for the positivity threshold of the diameter variation with a sensitivity of 90% on the observed data. To miss more than 10% of pain episodes was judged unacceptable and justified the choice of a sensitivity of 90%. Correlation between the COMFORT-B scores and the diameter variation was estimated using the coefficient of Spearman. All the statistical analysis was carried out using the R statistical software, version 4.0.3.

## Results

**Fig. 1:**
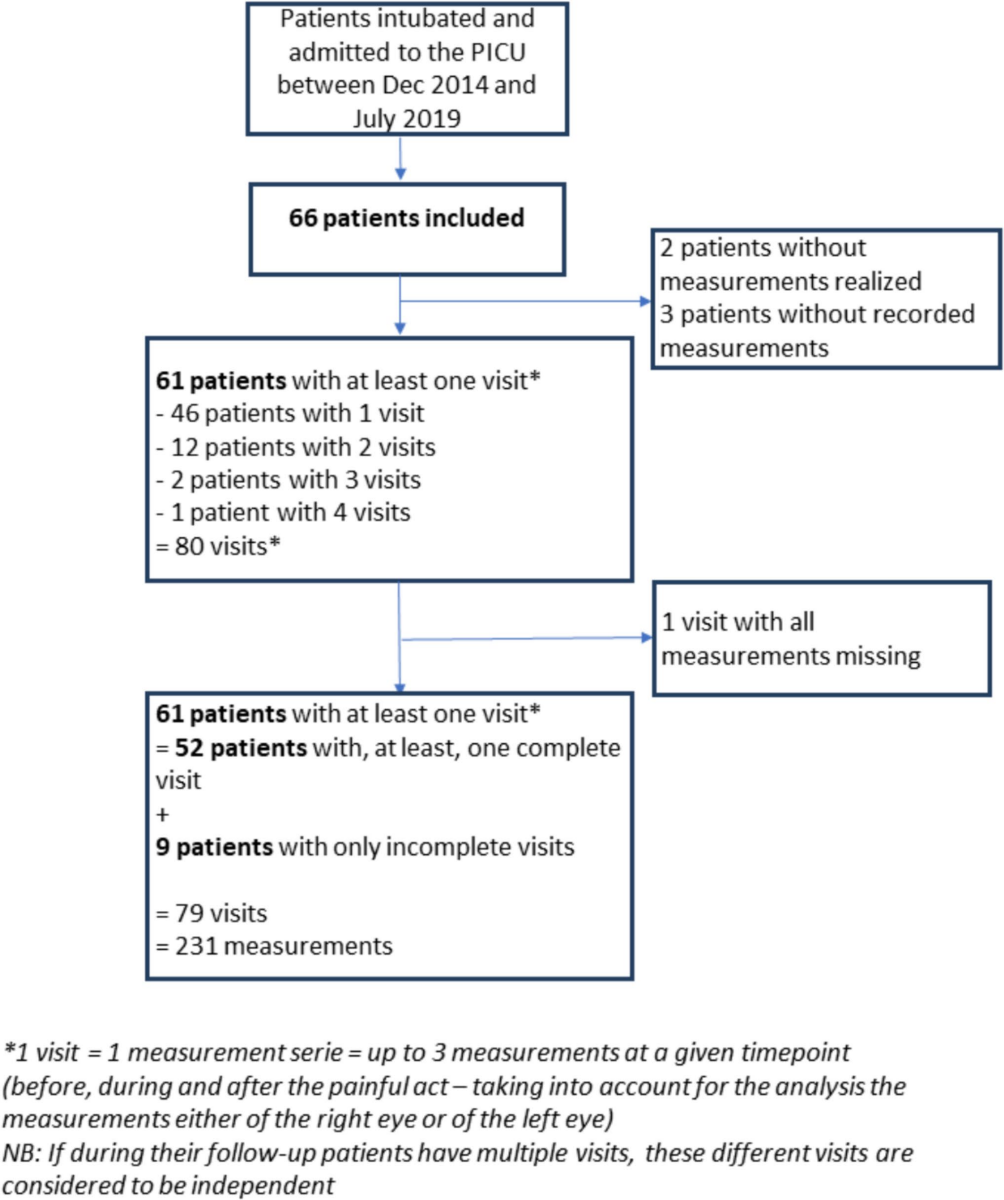
Flowchart of the study

Sixty-six patients were included between December 2014 and July 2019. A total of 61 patients were included in the analysis since three had no recorded measurements and measurements could not be performed in two others. Among these 61 patients, 52 had at least one complete measurement series (i.e., before, during, and after tracheal suctioning), while 9 had only partial sequences (with a maximum of two measurements). The study flowchart is presented in Fig. 1. A total of 80 measurement series were performed, but data were missing for one of them. At least one measurement was available for 79 series, resulting in a total of 231 individual measurements.

No adverse events occurred during the study’s procedures.

Among the children included, 47.5% were girls. The median age was 4 years (2.8–9.2), and the median weight was 16 kg. The most common reasons for hospitalization were trauma (31%), respiratory distress (18%), and postoperative care (13%). The most frequently administered drugs at the time of tracheal suctioning were midazolam (79%) and sufentanil (80%); 40% of patients also received ketamine. Baseline characteristics at inclusion are summarized in Table 1. Characteristics of patients with failure for all measurements or incomplete measurements are available in Supplementary (Tables S1 and S2).

**Table 1:** Patients’ initial clinical characteristics (patients for whom at least one measure is available)

	*N* = 61 patients
Age, years, median (Q1–Q3)	4.0 (2.8–9.2)
Weight, kg, median (Q1–Q3)	16.00 (13.00–32.00)
Sex, female, no. (%)	29 (47.5)
Reason of admission, no. (%)	
Respiratory distress	11 (18.0)
Sepsis/septic shock	1 (1.6)
Kidney failure	6 (9.8)
Trauma	19 (31.1)
Postsurgery	8 (13.1)
Other	16 (26.2)
Invasive ventilation duration, hours, median (Q1–Q3)	112 (47–242)
Sedatives at the time of the measure^*^, no. (%)	*N* = 80 series of measurements
Midazolam	63 (78.8)
Sufentanil	64 (80.0)
Ketamine	32 (40.0)
Propofol	8 (10.0)
Other	1 (1.2)

^*^N = 80 corresponds to the number of series of measures (one patient having potentially several series)

**Fig. 2:**
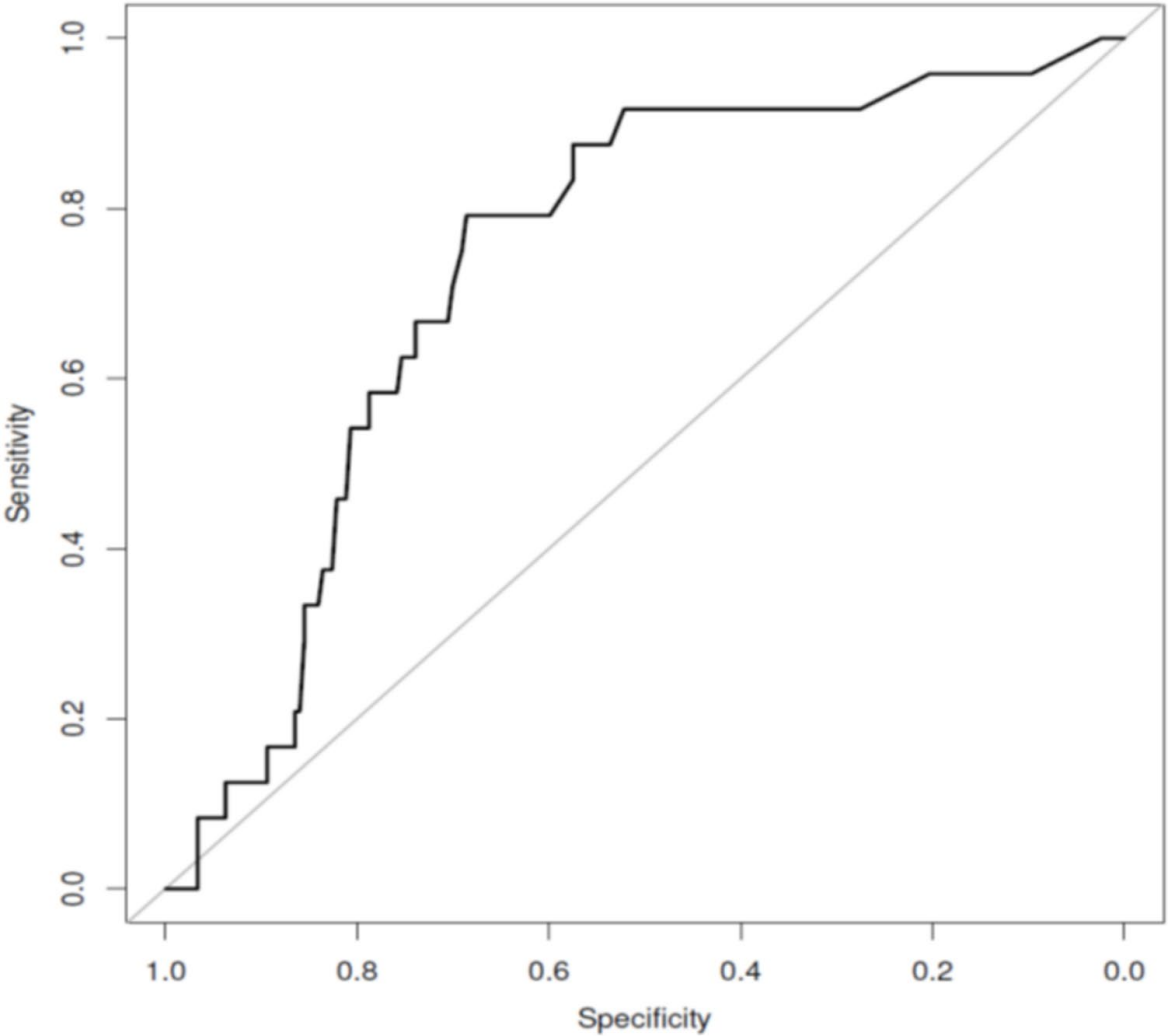
ROC curve of the variation in pupillary diameter (maximal diameter–minimal diameter)

The performance of pupillary diameter variation in pain assessment is shown in Fig. 2. The area under the curve (AUC) was estimated at 73.6% [95% CI 61.1–82.6].

Threshold determined to ensure a sensitivity test of 90% was an absolute variation of 0.145. The specificity for this threshold was estimated at 52.2%.

**Fig. 3:**
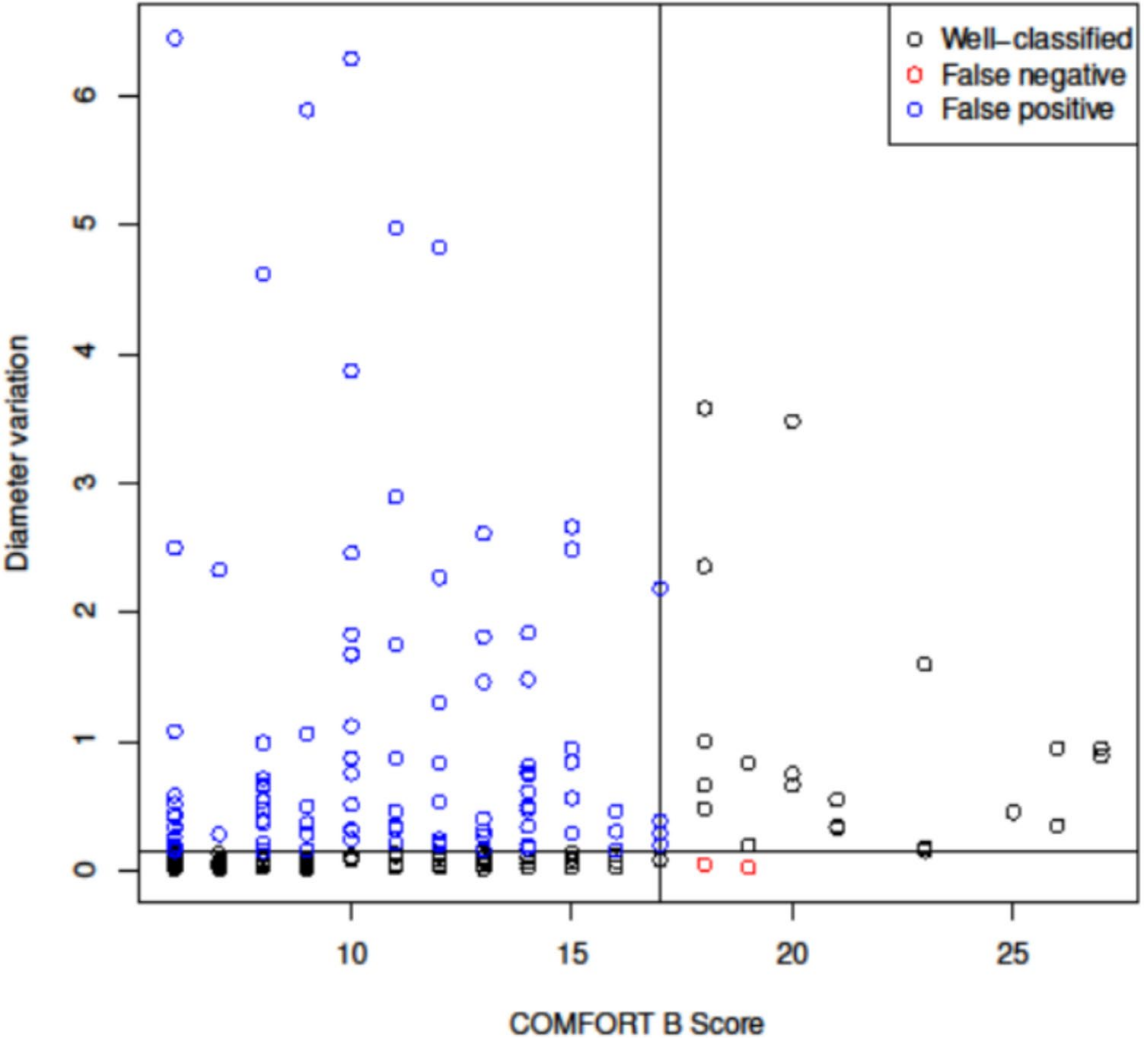
Scatter plot of pupillary diameter variation according to COMFORT-B score. The vertical black line corresponds to a COMFORT-B score of 17. The horizontal line corresponds to a diameter variation of 0.145 (the threshold defined to ensure a sensitivity of 90%). Each point represents an individual measurement: Black points indicate well-classified observations, red points indicate false negatives, and blue points indicate false positives

A weak correlation was found between the maximal pupillary diameter variation and the COMFORT-B score in children hospitalized in PICU (Spearman correlation coefficient 0.395, *p* < 0.05) (Fig. 3).

Pupillary diameter variation was greater in patients who were in pain or discomfort. Pupillary diameter was larger in children with a COMFORT-B score > 17 (Table 2). In our study population, pain prevalence was 10.4%. With this prevalence, at the identified threshold of an absolute variation of 0.145, with a sensitivity of 90% and a specificity of 52.2%, the negative predictive value was 99.98%.

**Table 2:** Description of pupillary diameter variation according to COMFORT-B and the time of measure

	Global *N* = 231 measures		Before pain *N* = 78 measures		During pain *N* = 79 measures		After pain *N* = 74 measures	
	COMFORT-B > 17	COMFORT-B ≤ 17	COMFORT-B > 17	COMFORT-B ≤ 17	COMFORT-B > 17	COMFORT-B ≤ 17	COMFORT-B > 17	COMFORT-B ≤ 17
Number of measures, no. (%)	24 (10.4)	207 (89.6)	4 (5.1)	74 (94.9)	16 (20.3)	63 (79.7)	4 (5.4)	70 (94.6)
Pupillary diameter variation, mm, median (Q1–Q3)	0.66 (0.34–0.95)	0.13 (0.05–0.47)	0.72 (0.41–1.58)	0.10 (0.05–0.39)	0.61 (0.31–0.91)	0.30 (0.11–0.95)	0.71 (0.58–1.15)	0.10 (0.04–0.31)

Use of video pupillometry: the proportions of complete, partial, and failed measurements are summarized in Table 3. Initial inclusions allowed us to identify that video pupillometry could not be performed in infants under 5 kg, as the device was not adapted to the ocular size of these children.

**Table 3:** Complete measurements/partial measurements/failed measurements

Measurements (at each time) *n* = 61 patients	Complete measurements (3 measure per eye) *n* = 52	Partial measures (at least one failure of measure) *n* = 9
Age (years), median (Q1–Q3)	4.2 (3.1–9.3)	2.2 (0.5–4.5)
Weight (kg) median (Q1–Q3)	16.8 (13.6–33.5)	14.0 (6.7–21.0)
COMFORT-B > 17 (at least one time)	8 (15.4%)	7 (77.8%)

Children with at least one measurement failure were the youngest patients included, with a median age of 2.2 years (0.5–4.5), compared to 4.2 years (3.1–9.3) in those without failure, and had lower body weight (14 kg vs. 16.8 kg).

Two complete failures were reported: one in an infant of 4 months of age and one in a waking patient in whom measurement with the device was not feasible. Regarding partial failures, 77.8% (7/9) of children for whom at least one measurement could not be performed had at least one COMFORT-B score > 17 during the measurement period, indicating that these patients were more likely to be awake or agitated. Conversely, among children with complete measurements, only 15.4% (8/52) had at least one COMFORT-B score above 17. Failures are summarized in Table 3.

## Discussion

Pupillometry has demonstrated its value in assessing pain in various settings, including evaluating pain in adult intensive care units, and in pediatric anesthesia [9, 10, 23, 24]. Our study aimed to explore the use of pupillometry specifically in PICU to assess analgesia in patients intubated and under sedation.

Despite an area under the ROC curve of the diameter variation equal to 73.6% [95% CI 61.1–82.6], our findings revealed a weak correlation between the COMFORT-B scale and changes in pupil diameter in patients experiencing pain. To ensure a sensitivity of 90% (in order not to miss children in pain), a diameter variation threshold of 0.145 was considered but it corresponded to a specificity of only 52%. This specificity is too low to support the use of this threshold in practice. This lack of correlation contrasts with results observed in the operating room.

Our study suggests that performing pupillometry in PICU is feasible, with 66% of success or complete measures. The main difficulties arose in younger children and those weighing under 5 kg, likely due to the device’s incompatibility with small orbital size. This observation is consistent with the findings of Tosi [25] with 20 children aged 6 months to 15 years, where none were less than 5 kg.

Partial or unsuccessful measurements were also caused by patient alertness during the assessment. Technical PICU-specific factors (e.g., fluid overload, prone positioning) also affected the feasibility of pupillometry and must be considered when evaluating its potential role in pain assessment in PICU patients. Several factors specific to critically ill patients (e.g., delirium, hypoxia, hypercapnia, or neurological disorder) may make it even more difficult to distinguish true pain. They may interfere with pupillary reflexes [34], possibly contributing to the absence of correlation between pupillary variation and COMFORT-B scoring in our study and explaining the differences observed with studies performed in operating room.

To our knowledge, this is the first study aiming to evaluate the performance of pupil diameter variation to assess pain in sedated PICU patients. We performed a total of 231 measurements, including 52 complete series, the largest pediatric cohort in terms of both measurement number and patient inclusion [10, 25].

The standardization of the painful procedure across all patients reinforces the reproducibility of our study compared with others where painful stimuli were not standardized [25]. The reproducibility of tracheal suctioning, however, was not perfect and might vary in terms of the number of saline instillations, duration of the procedure. Nevertheless, this procedure has been used in studies to assess pain using a similar design (before, during, and after the act) and was considered to induce a reproducible pain stimulus [26, 27]. We chose tracheal suctioning as the painful stimulus to avoid inflicting additional pain and to assess pupillary responses in the most clinically relevant context. Independent assessment of pupillometry and COMFORT-B scoring by two different observers helped limit measurement bias.

Pupillometry shows good sensitivity for detecting nociceptive responses in sedated patients but remains limited in its ability to discriminate pain intensity. Considering a threshold of 0.145 for pupil diameter variation, which yields high sensitivity but low specificity, the technique demonstrates strong detection capability while remaining limited in its ability to discriminate between nociceptive and nonnociceptive stimuli. Furthermore, the NPV of the maximal pupillary diameter variation indicates that the pupillometer could be a useful tool to rule out pain in noncommunicating children in the PICU.

Regarding sedation in PICU, it remains very challenging to standardize, due to common pharmacologic tolerance [19, 28, 29], making sedation management more complex. The most widely used sedatives were midazolam and sufentanil as first-line agents. In some cases, ketamine was added to overcome this issue and maintain adequate sedation. Pupil dilation in response to painful stimuli persists under ketamine (1 mg/kg IV) [30], which led us to include patients sedated with ketamine. Additionally, propofol may also exert a dose-dependent effect on the pupil [31], which may have further complicated correlation analyses with the COMFORT-B scale. Sedation duration was not accounted for in our analysis. Prolonged sedation may have blunted pupillary responses to pain, as previously described [13].

No correlation was observed with the validated COMFORT-B scale. One possible explanation is the limited specificity of the COMFORT-B scale, which assesses multiple components of the pain response, including anxiety, sedation, and nociception [2, 3]. In contrast, pupillometry strictly reflects nociception through the pupillary dilation reflex, including in anesthetized patients.

Moreover, several items in the COMFORT-B observational scale rely on subjective assessments, such as vigilance, calmness, muscle tone, and facial expression. The subjective nature of these variables reduces the reliability of observational scales, even when considered as “gold standard,” as previously suggested in studies assessing BIS monitoring [32, 33].

The lack of correlation may be explained, by specific factors related to PICU: the COMFORT-B scale itself, characteristics of sedation in pediatric patients, and presence of multiple confounding factors in the ICU that may interfere with pupillary diameter.

Nevertheless, COMFORT-B and clinical parameters such as blood pressure or cardiac frequency have also some limitations and sedation is very challenging to assess accurately, whatever the tool is. Then, we suggest that pupillometry could be integrated into a multimodal pain assessment strategy, as a point-in-time pain evaluation and may help identify certain painful stimuli in sedated critically ill children.

Further studies are needed to better define the role of pupillometry in PICU, particularly comparative strategy studies, according to the literature review by Mondardini published in 2023 [34].

Our study has some limitations as it was a single-center study, conducted in a unit where a nurse-driven sedation-analgesia protocol is in place [35], providing optimized pain management. However, complete standardization of sedation is impossible, as optimal sedation levels vary with underlying disease severity and the need for invasive procedures.

Also, our study lack power as we were not able to complete the expected number of participants after 5 years and stopped the study after 61 patients were included.

Pain and sedation are very complex to distinguish in PICU, regardless of the tool used. Based on our findings, pupillometry could represent an additional tool for pain assessment in critically ill children. The use of pupillometry in the PICU setting may contribute to a multimodal approach to pain assessment, as no current method offers both high sensitivity and high specificity. Pupillometry also appears useful in assessing children sedated with ketamine. Its role in the overall pain management strategy in this specific population should be further clarified by additional research. The youngest children remain the most challenging population to manage with sedation and analgesia.

## Supplementary Information

Below is the link to the electronic supplementary material.

ESM 1 Supplementary Material 1 (DOCX 98.3 KB)

## Data Availability

All data are available upon reasonable request from the corresponding author.

## Abbreviations

AUC: Area under the curve
BCa: Bias-corrected and accelerated
BIS: Bispectral Index
CI: Confidence interval
ICC: Intraclass correlation coefficient
PICU: Pediatric intensive care unit
PPI: Pupillary Pain Index
ROC: Receiver operating characteristic
STARD: Standards for Reporting Diagnostic Accuracy Studies
VAS: Visual Analog Scale
