# Supplementary material for: Pupillometry for pain assessment in noncommunicating children in the pediatric intensive care unit: a prospective accuracy study
Source: Eur J Pediatr. 2026 May 2;185(5):334. doi: 10.1007/s00431-026-07004-3 (PMC13135524; doi:10.1007/s00431-026-07004-3)
Supplement: Supplementary file 1 — Supplementary Material 1 (DOCX 98.3 KB) [file 431_2026_7004_MOESM1_ESM.docx]

Supplementary: ***Pupillometry for Pain Assessment in Non-Communicating Children in the Pediatric Intensive Care Unit: A Prospective Accuracy Study***


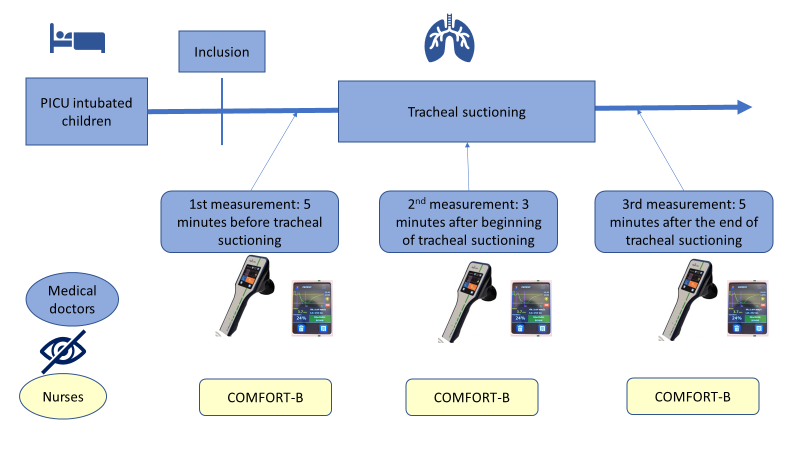


**Figure S1:** Timeline of measurements in PUPILLO-STUDY

Patients were included in the PICU. Each included patient underwent three measurements per series. A given patient could be included in multiple series. Pupillometer measurements were performed 5 minutes before, during, and 5 minutes after tracheal suctioning by a dedicated physician. At the same time, nurses assessed the COMFORT-B scale. Physicians and nurses were blinded to each other’s assessments. PICU = Pediatric Intensive Care Unit.

Table S1: Patients with failure of all measurements

| Patient | Sex | Age | Weight (kg) |
| --- | --- | --- | --- |
| 01-10 | M | 3 y | 12.5 |
| 01-14 | F | 2 m | 4.1 |

M=Male, F= Female; m=months, y= years

Table S2: Patients with incomplete measurements

| Patient | Sex | Age | Weight (kg) | Time of failure |
| --- | --- | --- | --- | --- |
| 01-01 | F | 2 m | 4.8 | After |
| 01-02 | M | 4 m | 6.7 | During and After |
| 01-03 | F | 4 y | 21 | After |
| 01-06 | M | 5 m | 4 | After |
| 01-08 | M | 2 y | 14 | After |
| 01-15 | M | 13 y | 40 | During |
| 01-26 | F | 4 y | 14 | After |
| 01-49 | M | 16 y | 58.5 | Before |
| 01-64 | M | 1 y | 9 | During |

M = male, F = female; m = months, y = years. “Before” indicates that the first measurement (before tracheal suctioning) was not performed; “during” indicates that the measurement during tracheal suctioning was not performed; and “after” indicates that the last measurement (after tracheal suctioning) was not performed.
